# Enhancing stress measurements accuracy control in the construction of long-span bridges

**DOI:** 10.1038/s41598-024-61873-y

**Published:** 2024-05-14

**Authors:** Alvaro Gaute-Alonso, David Garcia-Sanchez, Óscar Ramón Ramos-Gutierrez, Vasileios Ntertimanis

**Affiliations:** 1 Instrumentation and Dynamic Analysis of Structures Group (GiaDe), University of Cantabria, Santander, Spain; 2 TECNALIA Basque Research and Technology Alliance (BRTA), Derio, Spain; 3 Department of Structural and Mechanical Engineering, University of Cantabria, Santander, Spain; 4 Department of Construction, Environment and Geomatics, University of Zurich, Zürich, Switzerland

**Keywords:** Structural health monitoring systems, Load cells, Evaluation of pre-stressing losses, Cable-stayed cantilever technique, Arch bridges, Engineering, Materials science

## Abstract

This paper introduces new contributions for construction procedures designed to enhance the robustness and precision of stress control in active anchorage and short presetressing units for long-span bridges, particularly addressing potential technical risks. The primary focus is on optimizing stress management for bridge stays, suspension cables, and short prestressing units by emphasizing a unified parameter: stress. The contributions of this research encompass (1) the introduction of advanced load cells for stress control in active anchorages and (2) the implementation of a novel synchronized multi-strain gage load cell network for short prestressing units, crucial in situations where prestressing losses can attain significant magnitudes. To validate these advancements, the authors present (3) a practical experience and results obtained from applying these methodologies in monitoring the structural response during the construction of the Tajo Bridge using the cable-stayed cantilever technique.

## Introduction

In the design of long-span bridges, cable-stayed or suspension solutions are commonly used. These solutions enabled to overcoming the 1000 m span barrier in the twentieth century^1^ and the 2000 m span barrier in the twenty-first century^2,3^. Damage associated with fatigue and/or corrosion^4,5^ caused by dynamic loads, such as cyclic loads from traffic or wind, is the main factor conditioning the durability of stayed or suspended cables. Temporary suspension cables for the construction phase present the same problems.

The most important parameter for assessing the influence of fatigue and corrosion damage to cables in service is the axial stress. It is, indeed, the key parameter to be controlled for safety condition assessment of stay and suspension cables in service bridges^6,7^, during construction operations^8,9^ and also dismantling procedures^10^. Various devices have been developed for the direct and indirect measurement of stress in bridge cables. Among the devices used for direct stress measurement are load cells^11,12^, fibre optic Bragg grating sensors^13^ or elasto-magnetic strain sensors^14,15^. While the most common indirect methodology for rapid stress assessment in bridge cables is the vibrating wire technique^16–18^.

Another important parameter to be monitored during construction operations is the structural response of the auxiliary elements used for their construction, such as the temporary stay-cable towers used in bridges built using the successive cable-stayed cantilever technique. In the configuration of these auxiliary towers, it may be common to embed them in the base by means of a prestressed connection, using short prestressing units. In the particular case of short prestressing units, it is well known that these structural elements experience high instantaneous prestressing losses, so it is necessary to monitor their prestressing stress^19–21^, as well as the variation of this stress over time. This monitoring makes it possible to verify that the prestressing unit is working as stipulated in the project and to take preventive measures in case of detecting possible anomalies^22,23^.

## Monitoring experiences in long-span bridges monitoring

### Review of recent experiences in the monitoring of bridge stays

#### Traditional unidirectional strain gauges on one of the strands composing the stays

Typically, each of the strands that make up bridge stay cables is composed of 7 high tensile steel wires stranded together^24,25^. To characterise the existing tension in the cable, one of the strands is instrumented by installing 2 strain gages connected to each other by means of a complete electronic Wheatstone bridge assembly^26–28^. The deviation of the directrix of the instrumented wire with respect to the strand directrix makes it necessary to establish a correlation coefficient between the deformation experienced by the wire and the deformation experienced by the strand. In order to obtain an identical tension in all the strands that make up the stay cable, it is common to use the "Isotensioning" technique^29^.

#### Indirect stress measurement based on vibration frequency and mass

This technique allows measuring the stress in bridge stays by characterising their vibration frequency and mass^30–32^. The low bending stiffness (EI) of bridge stays allows estimating the stress in these structural elements from the relationship expressed in Eq. (1). The records of the acceleration experienced by the bridge stays are analysed by applying the fast Fourier transform^33–35^, which makes it possible to obtain the natural frequencies of the stays and, therefore, to calculate their axial force.

1 $$f=\frac{u}{2\cdot L}\cdot \sqrt{\frac{T}{m}} $$

where: T = axial force in the stay; u = mode of vibration considered; f = frequency of vibration corresponding to mode "u"; L = length of vibration of the stay; m = mass per linear meter of the stay.

**Figure 1:**
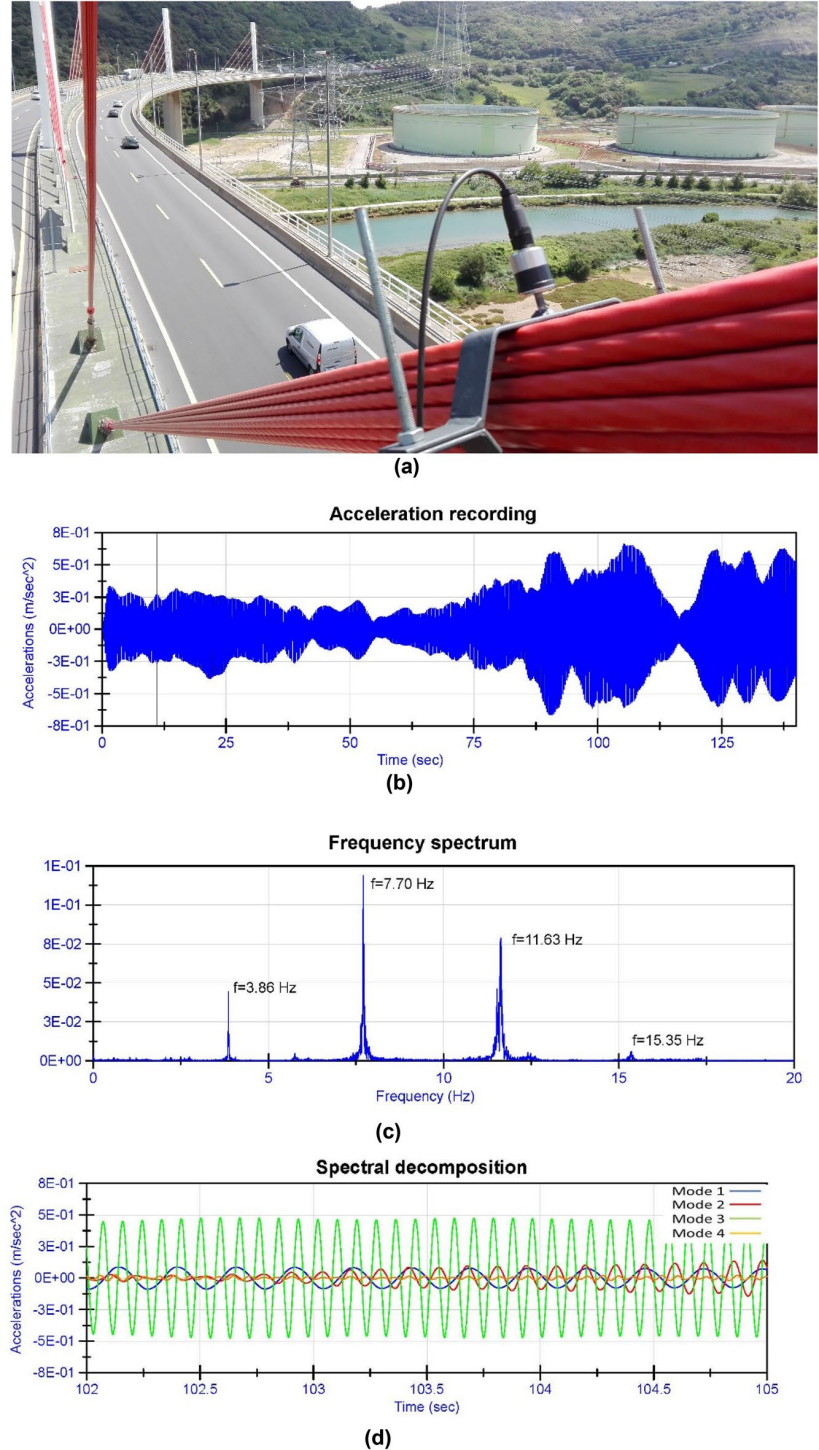
Obtaining the stress in a stay during structural repair work on a cable-stayed bridge: (**a**) installation of the accelerometer on the bridge stay; (**b**) recording of accelerations in the stay; (**c**) frequency spectrum of the bridge stay; (**d**) spectral decomposition of the recording of accelerations in the bridge stay.

The use of this technique for the monitoring of stresses in bridge stays can be carried out at any stage of the construction process or the service life of the structure. Furthermore, this technique allows the absolute value of the tension in the stay cable to be recorded, irrespective of the construction or service phase at which the sensor is installed. In addition to its use for structural monitoring of the tension in bridge stays over time, this technique is ideal for the determination of the existing tension in stays during inspection, rehabilitation and structural repair work on bridges and structures^10,36^. In Fig. 1, the authors show an example of the use of this technique during structural repair work on a cable-stayed bridge in northern Spain.

### The Tajo Bridge experience

**Figure 2:**
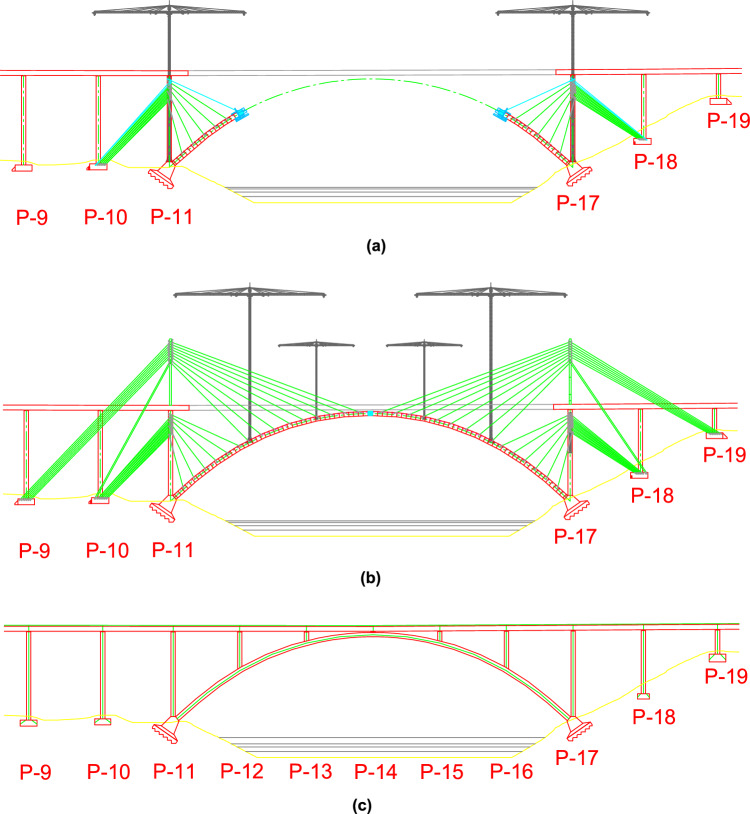
Construction process of the central arch of the Tajo Bridge: (**a**) provisional stay of the half-arches from piers 11 and 17; (**b**) provisional stay of the half-arches from the provisional stay towers; (**c**) keystone closure and dismantling of the provisional stay system.

The Tajo Bridge is a unique high-speed infrastructure designed by Carlos Fernández Casado's design office and built by ADIF (Spanish Railway Infrastructure Administration)^37^. The bridge is located at the mouth of the Tajo River at the Alcántara reservoir in the province of Cáceres, Spain. Its design and construction have been carefully planned to meet high-speed standards, guaranteeing efficiency and safety through advanced engineering and offering modern aesthetics. The bridge has a total length of 1488 m, with a span distribution of 45 m + 9 × 60 m + 57 m + 6 × 54 m + 57 m + 7 × 60 m + 45 m^32,33^. The 6 × 54 central spans are supported by an arch that spans the Tajo River with a total span of 324 m between supports. At the time of its construction, between 2012 and 2016, its central span was to make it the high-speed bridge with the longest concrete arch span in the world, only to be surpassed by the Almonte Bridge, with a central arch span of 384 m^38^. The arch was built using the technique of successive cable-stayed cantilevers. Each of the half arches consisted of a total of 46 segments, plus the arch keystone, and was temporarily suspended during construction by a total of 15 families of 4 stays (2 suspension stays and 2 restraint stays). The half arches were cantilevered from the foundations of piers 11 and 17 of the bridge, and every 3 segments a temporary stay was placed to suspend the existing cantilevered half arch from the piers, in the case of the first 6 families of stays (segments 1–20), or temporary stay towers in the case of the other 7 families of stays (segments 21–46) (see Fig. 2). To experimentally characterize the structural response of the central arch span, the authors designed a Structural Health Monitoring System (SHMS) composed of the following elements: (1) Management and Unification System with the project (M&USP) composed of the execution project databases elaborated by the project management of Carlos Fernandez Casado (https://www.cfcsl.com/), authors of the design and construction project of the Tajo Bridge; (2) Sensor System (SS) composed of a total of 114 sensors (Table 1): 30 load cells in the suspension cables + 8 load cells in the anchorages of the stay-cable towers + 40 unidirectional strain gauges in the reinforcement of the half-arches + 24 temperature probes + 16 clinometers + 3 anemometers + 3 accelerometers; (3) Data Acquisition and Processing System (DA&PS) composed of 2 modular Central Data Acquisition Systems (CDASs) model NI PXIe-1078, one on each bank of the Tajo river, connected by 2 point-to-point Wi-Fi antennas + 9 NI Model PXIe-4330 strain gauge Data Acquisition Units (DAUs) + 2 DAUs for voltage signals model NI PXI-6224 + 2 DAUs for Resistance Temperature Detector (RTD) signals model NI PXIe-4357 + 1 DAU for the accelerometers model NI PXIe-4492; (4) Data Management and Processing System (DM&PS) (DM&PS), consisting of a series of routines designed and programmed by the authors, whose purpose is the routing of variables, transmission, visualisation and storage of data, as well as the establishment of early warning systems fed by the DM&PS; (5) Structural Safety and Assessment System (SS&AS) composed of all entities involved in the construction of the Tajo bridge: the site technical team, the technical office, the site management and the project management office. This subsystem was responsible for monitoring the data provided by the instrumentation and for the comparative analysis of these data with respect to the project's theoretical data. The result of this comparative analysis generated an update of the M&USP databases and fed the SHMS^39,40^.

**Table 1:** Description of the Tajo bridge sensor system.

Sensors	Functionality
Load cells	Stress on the suspension stays (30 units)
	Stress in the prestressing units of the stay-cable towers (8 units)
Extensometers	Stress in the reinforcement of semi-arches (32 units)
	Stress in the starting reinforcement of the stay-cable piers (8 units)
Temperature probes	Vertical thermal gradient in the semi-arches (16 units)
	Thermal gradient in stay-cable piers and towers (8 units)
Clinometers	Deformation of the semi-arches (14 units)
	Twisting at the head of stay-cable piers and towers (2 units)
Anemometers	Wind incident in the north half-arch advance segment (1 unit)
	Wind incident at the head of the stay-cable towers (2 units)
Accelerometers	Stress on the suspension stays (1 unit)
	Acceleration on the suspension stays (1 unit)
	Acceleration on the semi-arches (2 units)

#### Monitoring of the deformation experienced by the reinforcement of the half-arches

**Figure 3:**
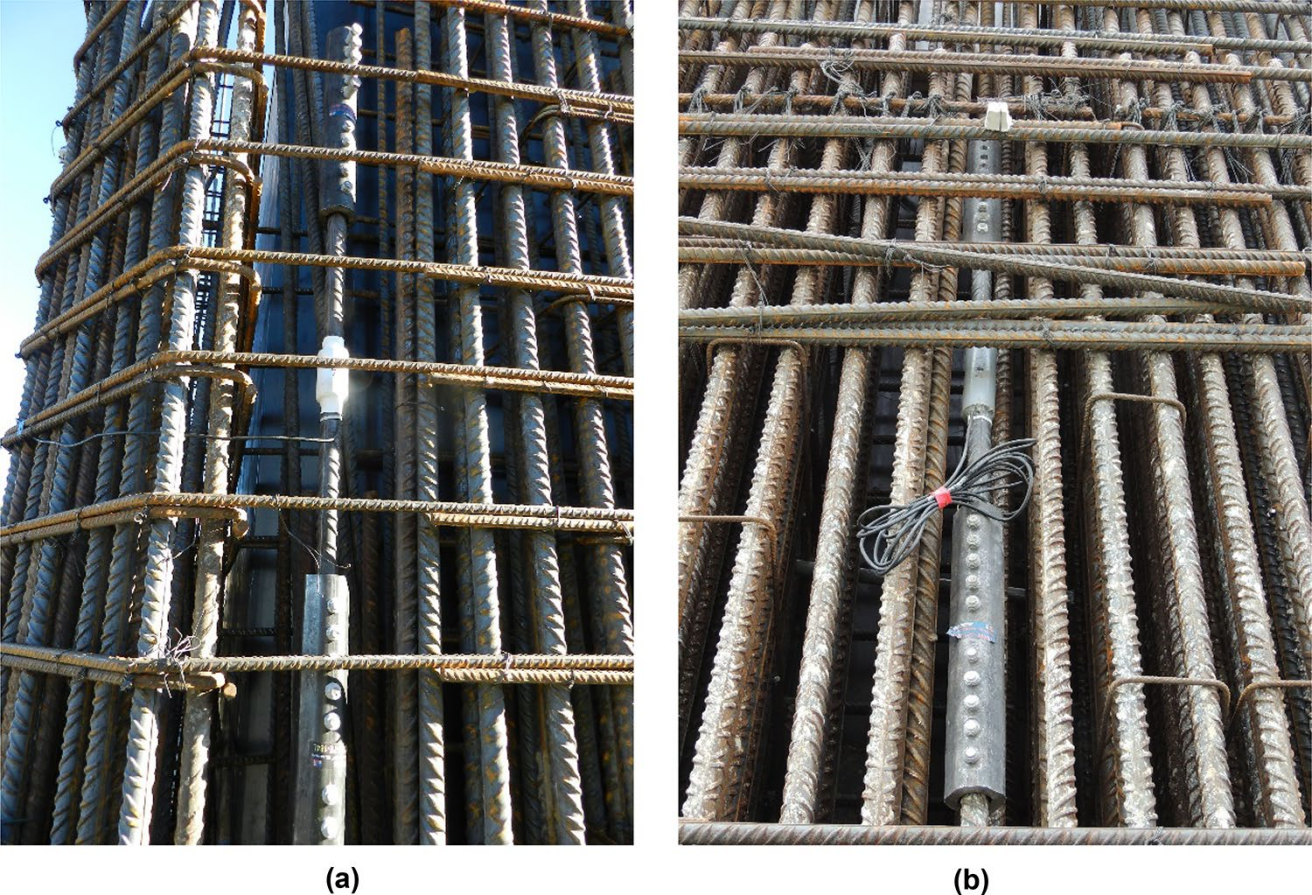
Extensometers in the passive reinforcement of the Tajo Bridge arch: (**a**) extensometer in the reinforcement of the stay piers; (**b**) extensometer in the reinforcement of the half-arches.

**Figure 4:**
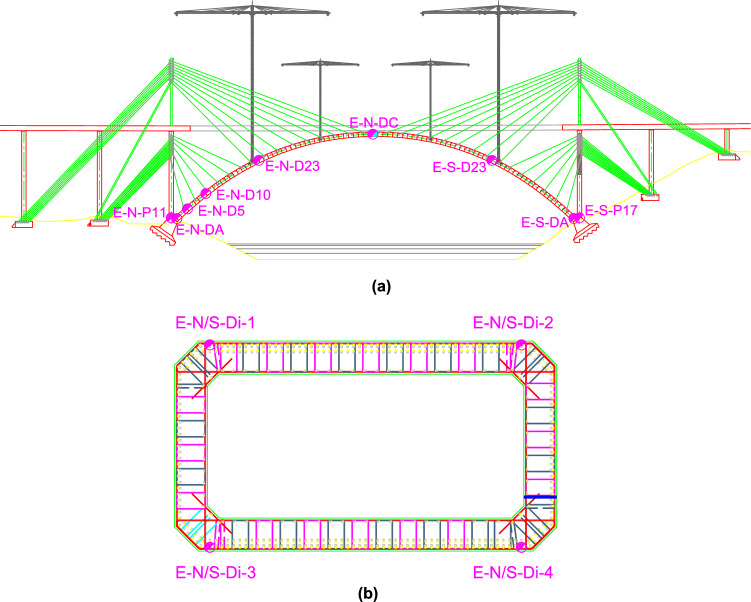
Instrumentation using extensometers: (**a**) identification of the instrumented segments; (**b**) standard arrangement of the extensometers in the structural section.

**Figure 5:**
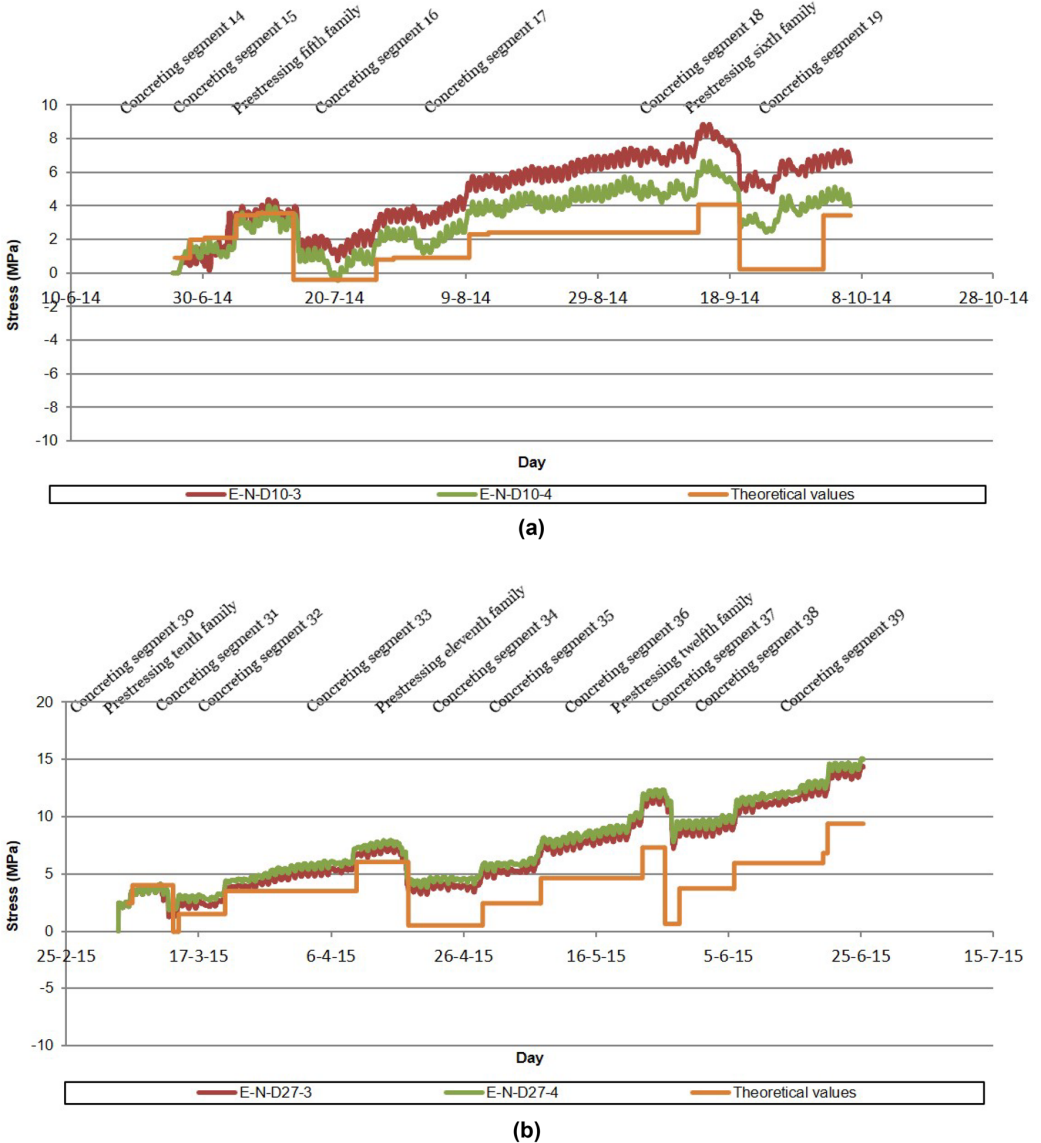
Monitoring of the stress in the segments of the half-arches: (**a**) evolution of the normal stress in segment 10; (**b**) evolution of the normal stress in segment 27.

It was considered necessary to control the stresses experienced by the different structural sections of the half arches and the stay piers during the construction of the semi-arches by means of the technique of successive cable-stayed cantilevers. To this end, the authors proposed the instrumentation of the longitudinal passive reinforcement in the following sections: (a) starting section of the stay piers (E-N-P11 and E-S-P17); (b) starting and kidney (segment 23) segments in both half-arches (E-N-DA, E-N-D23, E-S-DA and E-S-D23); (c) segment 5 and segment 10 in the north half-arch (E-N-D5 and E-N-D10); (d) closing keystone of the arch (E-N-DC). The instrumentation of these structural elements was carried out by installing a total of 40 longitudinal extensometers. These devices, designed and manufactured by the authors, consisted of a Ø32 passive steel bar, instrumented by two bi-directional strain gages connected to each other by a full Wheatstone bridge connection^26–28^, and connected to the longitudinal reinforcement of the arch and the piers above the arch starts by mechanical couplers (Fig. 3). Each section under study was instrumented with a minimum of 4 longitudinal strain gauges, one at each corner of the outer perimeter of the cross-section. This arrangement allows characterisation of the strain plane experienced by the structural section, due to a combination of possible axial, longitudinal bending and transverse bending stresses (see Fig. 4). The suspension of the half-arches of the Tajo Bridge by means of the families of stay cables allowed its cantilever construction without significant tensile normal stresses occurring in any structural section. The data analysis carried out at the SS&AS established a warning system for possible undesirable phenomena, such as the appearance of significant tensile stresses that could cause cracking of the upper slab of the arch box section due to excess negative bending stresses during the concreting of the successive segments, or cracking of the lower slab due to excess positive bending stresses caused by the stressing of the successive families of stay cables. Figure 5 shows the evolution of the normal stress experienced by the bottom slab of segment 10 (E-N-D10-3 and E-N-D10-4) and segment 27 (E-N-D27-3 and E-N-D27-4) of the north half-arch and its comparison with the theoretical design values reflected in the M&USP. These theoretical values correspond to the project values, provided by the engineering office Carlos Fernandez Casado (https://www.cfcsl.com/). The values provided by the sensors were initialised at the origin, so the stresses shown in the graphs in Fig. 5 correspond to absolute stresses. Positive values correspond to compressive normal stress values and negative values to tensile values. The format of the dates in the graphs corresponds to day-month-year.

#### Deformation monitoring of half-arches, stay piers and stay towers

**Figure 6:**
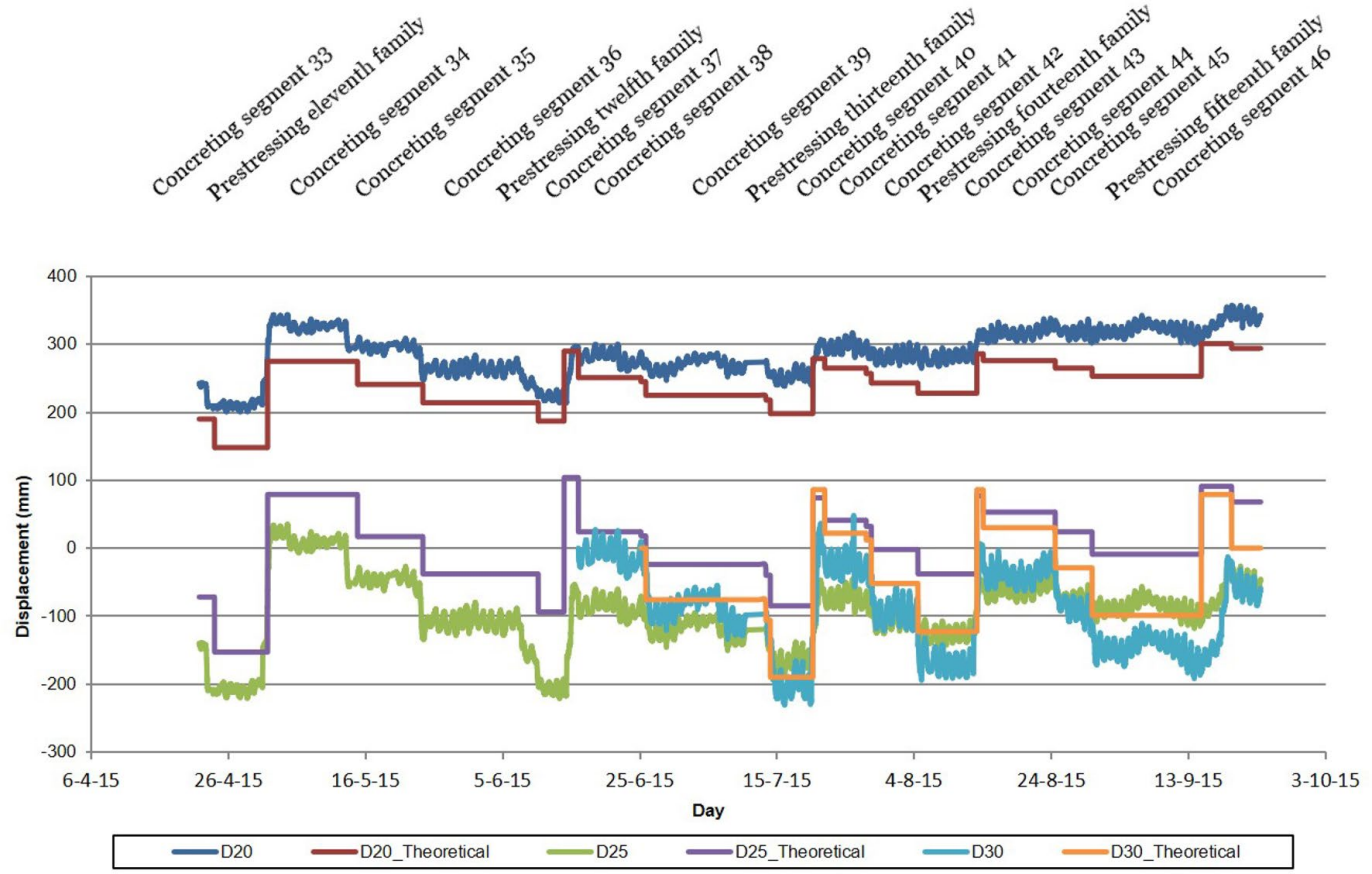
Monitoring of the deformation of the half-arches. Displacement of the instrumented segments of the northern half-arch.

It was proposed to monitor the deformations of the half-arches by means of clinometers embedded in the starting sections and segments 5, 10, 15, 20, 25, 30, 35 and 40 of the north half-arch, starting sections and segments 18, 25 and 33 of the south half-arch, and the closing segment in the keystone. The uniform distribution of the clinometers in the northern half-arch of the Tajo Bridge made it possible to obtain in real time the deformation experienced by this structural element by integrating the twists of the instrumented cross sections^41–43^ (Fig. 6). During the construction of the half-arches of the Tajo Bridge, the advancing segment could experience daily variations in height of the order of 20/30 mm due to daily increases in terms, decreases of up to 200 mm due to the cantilever concreting of a segment, and increases of up to 400 mm caused by the tensioning of a family of temporary stay cables. Figure 6 shows the deformations experienced by the segments near the kidney (segment 23) of the north half-arch, segment 20 (D20), segment 25 (D25) and segment 30 (D30), and their comparison with the theoretical design values, during the period of time coinciding with the concreting phases of segments 33–46 and the tensioning of stay cable families 11–15. Positive values correspond to an uplift of the cantilevered segment. Another important parameter during the construction process of the half arches was the twist experienced by the headers of the stay piers, during the concreting of the first 21 segments of each half arch and the tensioning of the first 6 families of stay cables, and of the provisional stay towers, during the rest of the construction phases up to the closure of the arch at the keystone. For the structural characterization of this parameter, the authors proposed the instrumentation of the headers by installing a high-precision clinometer.

#### Monitoring of the acceleration experienced by the northern half-arch

In order to experimentally characterize the possible occurrence of aeroelastic phenomena n the half-arches of the Tajo Bridge, the authors proposed the instrumentation of the north half-arch by installing a unidirectional accelerometer in segment 20 and a triaxial accelerometer in segment 36^44–46^. This instrumentation made it possible to dynamically characterize the behavior of the Tajo Bridge: (a) to obtain the frequencies associated with the fundamental modes of vibration of the northern half-arch in each of the phases of the construction process; (b) to record the accelerations experienced by the instrumented segments and to establish early alarms in the event of the possible appearance of undesired aeroelastic phenomena.

#### Monitoring of the thermal gradient in different structural sections and of the wind incident on the structure

**Figure 7:**
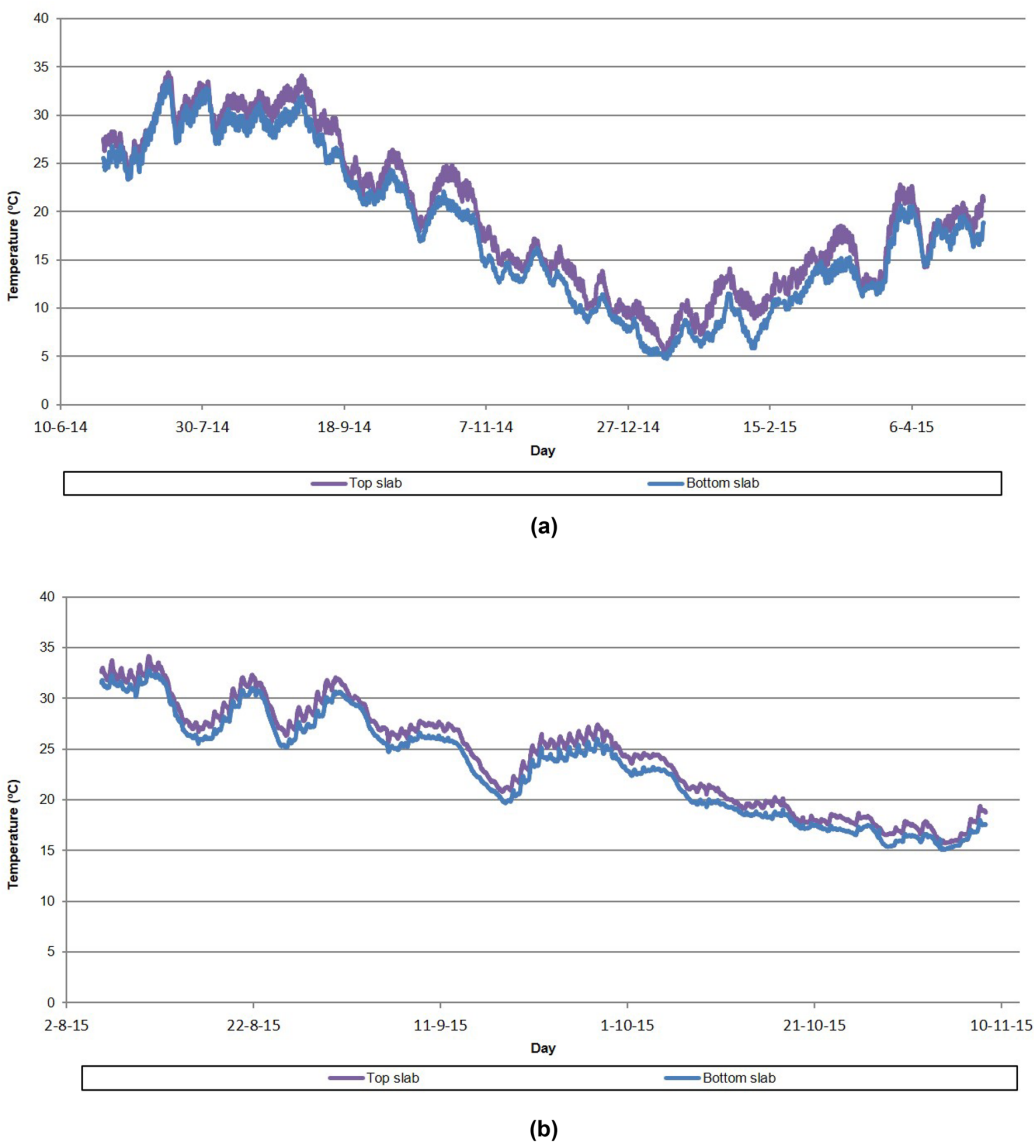
Monitoring of the vertical thermal gradient: (**a**) segment 10 of the northern half-arch; (**b**) segment 40 of the northern half-arch.

The evolution of the vertical thermal gradient has been followed in the starting section and segments 10, 25 and 40 of the northern half-arch, in the starting section and segment 25 of the southern half-arch, in the keystone closing segment, and in the starting and head sections of the provisional stay tower on the north bank. The characterization of the thermal gradient in the study sections is carried out by installing two temperature probes, each located at the center of gravity of the upper and lower slabs of the structural section (Fig. 7). By characterising only the vertical thermal gradient, only the influence of the thermal gradient on the deformation of the half-arches concomitant to longitudinal bending is obtained. If the engineering team responsible for the design and construction of the half-arches had considered characterising the influence on the transverse deformation, the horizontal thermal gradient would have had to be characterised. This could have been done by installing two additional temperature probes in each instrumented section, one at the centre of gravity of each web of the half-arches' cross-section. The characterization of the wind incident on the structure was resolved by the installation of 3 ultrasonic anemometers, 1 anemometer at the head of each temporary stay tower and a third device at the cantilevered front section of the north half-arch.

## New revisited monitoring systems for stress control. Results and Discussion based on the Tajo Bridge experience

### New load cells for active anchors

**Figure 8:**
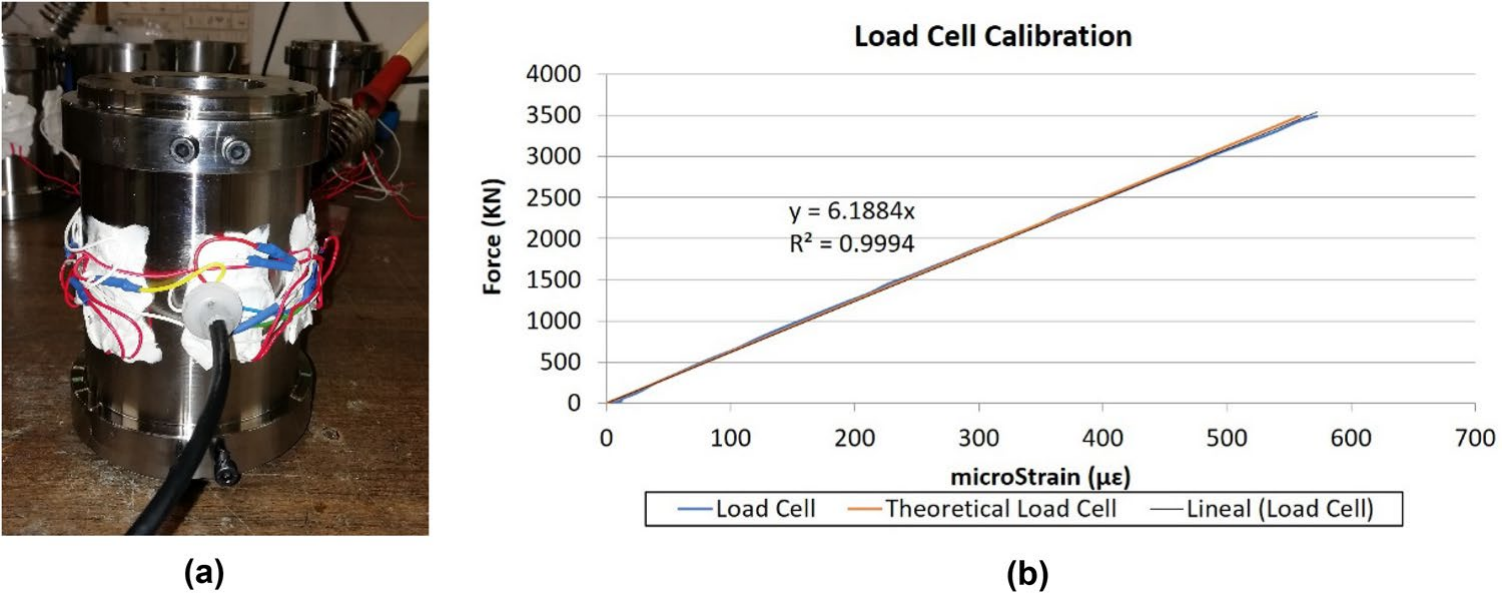
New load cells for active anchors: (**a**) instrumentation of the outer perimeter of the load cell centre ring; (**b**) load cell force/strain calibration curve.

**Figure 9:**
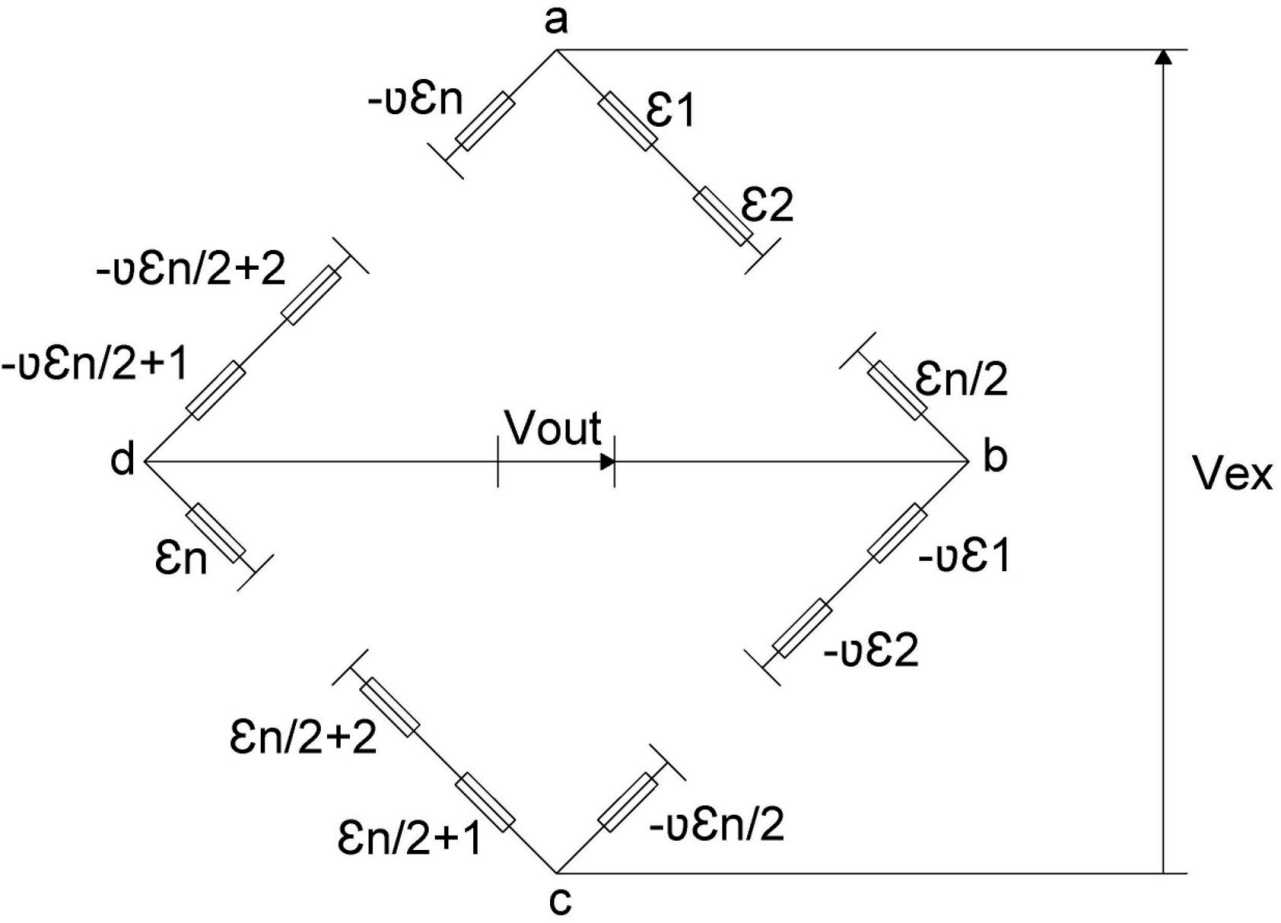
Electronic connection of the strain gages arranged around the outer perimeter of the central ring of the device.

As indicated above, the monitoring of the axial force transmitted by the bridge stays is one of the most important aspects in stress controlling the correct structural behavior of the bridge. This can be transferred to prestressed structures, where the monitoring of the axial force of the prestressing units is a fundamental support to control their correct structural performance. To this end, the authors set out to design and manufacture a device capable of performing this function. This device had to meet the following requirements: (1) accurately characterize the total axial force transmitted by the bridge stay or prestressing unit; (2) provide a robust solution for extreme environments, shocks and impacts; (3) provide direct measurement without the need for signal integrators. The device designed by the authors consists of a metal ring that allows the bridge stay or prestressing unit to pass through it. Thus, the prototype would be placed between the anchor plate of the bridge stay or prestressing unit and the distribution plate on the structure. The outer perimeter of the central ring of the prototype is instrumented by the uniform distribution of bidirectional strain gauges (Fig. 8) connected in series by a Wheatstone full-bridge electronic assembly^26–28^. Gauges aligned with the direction of the axial force (Ɛ_i_), the axis of revolution of the device, are connected in series on arms 1 ($\overline{ab }$) and 3 ($\overline{cd }$) of the Wheatstone bridge, while gauges aligned perpendicular to the direction of the axial force (μƐ_i_) are connected in series on arms 2 ($\overline{bc }$) and 4 ($\overline{da }$) of the Wheatstone bridge (Fig. 9). This instrumentation makes it possible to obtain the mean axial deformation of the metal ring, a measurement that is linearly proportional to the total axial force transmitted by the bridge stay or the prestressing unit (Eq. (2)). Figure 8 shows the electronic assembly of a load cell in which a total of "n" bi-directional strain gauges are arranged. The gauges on the active arms of the Wheatstone bridge, those aligned with the longitudinal axis of the load cell, are represented as "Ɛ_i_", while the gauges on the compensating arms of the Wheatstone bridge, those aligned perpendicular to the longitudinal axis of the load cell, are represented as "μƐ_i_". The bridge is supplied with a certain excitation voltage "V_ex_", a function of the number of gauges arranged in the load cell and the type of reading device or "DAU", and an output voltage "V_out_" is obtained which is directly proportional to the longitudinal deformation experienced by the load cell. In the load cells designed by the authors, the strain gauges located on the outer perimeter of the device are manually installed by the laboratory design engineers. This implies that there may be minimal deviations between the alignment of the bi-directional strain gauges and the alignment of the longitudinal axis of the device, resulting in a possible slight deviation between the value provided by the load cell and the result of Eq. (2). Therefore, it is always necessary to calibrate the load cells in the laboratory, this process is done by placing the load cell in a press and synchronising the load reading of the press and the strain reading of the load cell. The material of which the load cell body is made is normally steel, but any other material could be used taking into account its mechanical characteristics. As shown in Eq. (3), the yield strength of the material will define the outer diameter of the device. While, as shown in Eq. (2), the modulus of elasticity of the material will be the linear coefficient relating the force applied on the device to the product of the average strain of the strain gages and the effective cross-sectional area of the device.

2 $$ F = \mathop{{\int\!\!\!\!\!\int}\mkern-21mu \bigcirc} {\sigma \cdot d\Omega } = \frac{{\mathop \sum \nolimits_{1}^{n} \varepsilon_{i} }}{n} \cdot E_{a} \cdot \Omega_{c} $$

where: F = stress in the bridge stay; σ = normal stress; dΩ = area differential in the central ring; ɛ_i_ = normal strain in the ith strain gauge; n = number of strain gauges; E_a_ = modulus of elasticity of the steel; Ω_c_ = area of the central ring.

**Figure 10:**
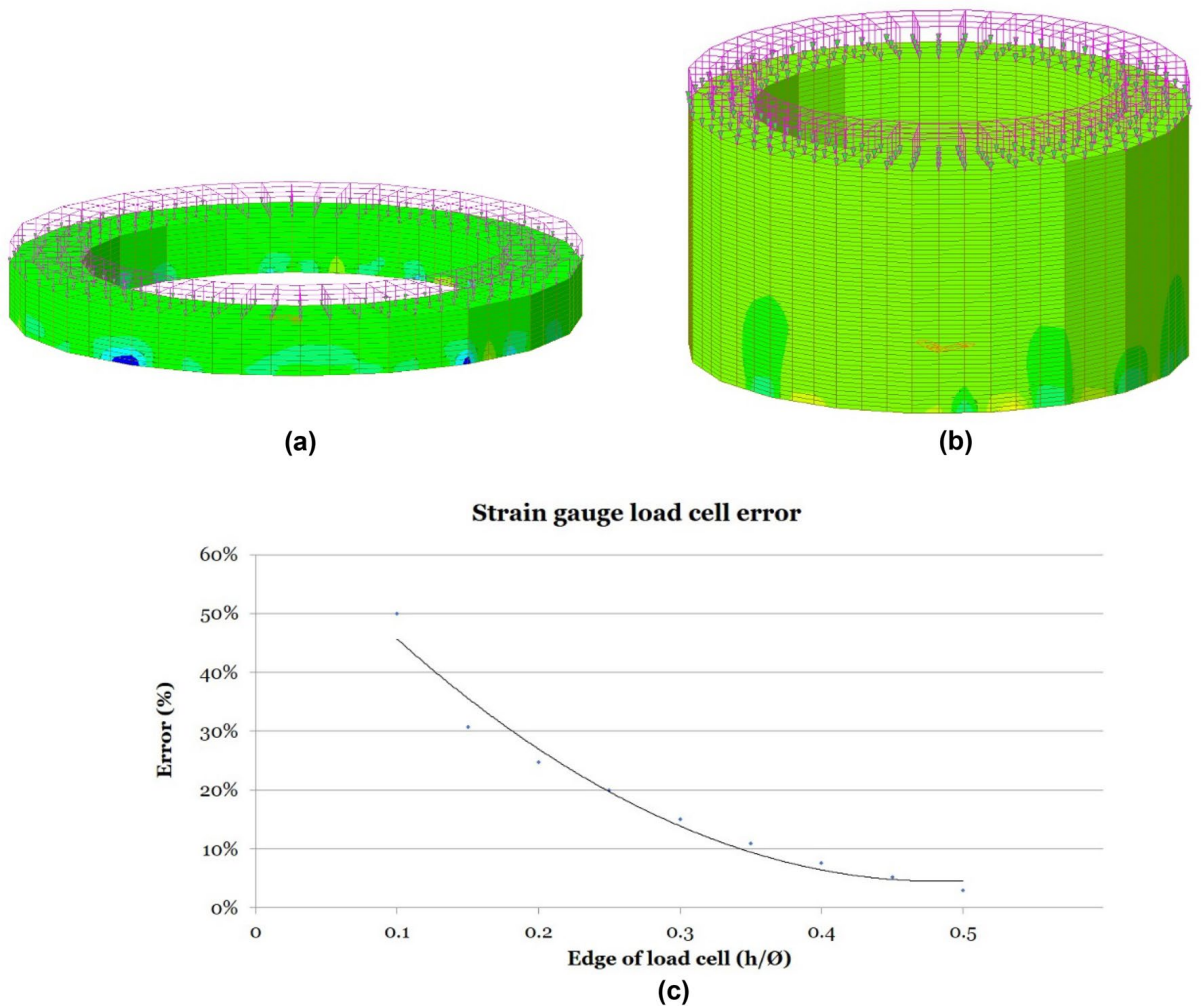
Parametric study for load cell edge optimization: (**a**) finite element model with h = ∅/10; (**b**) finite element model with h = ∅/2; (**c**) error evolution curve with edge/diameter ratio (h/∅).

**Figure 11:**
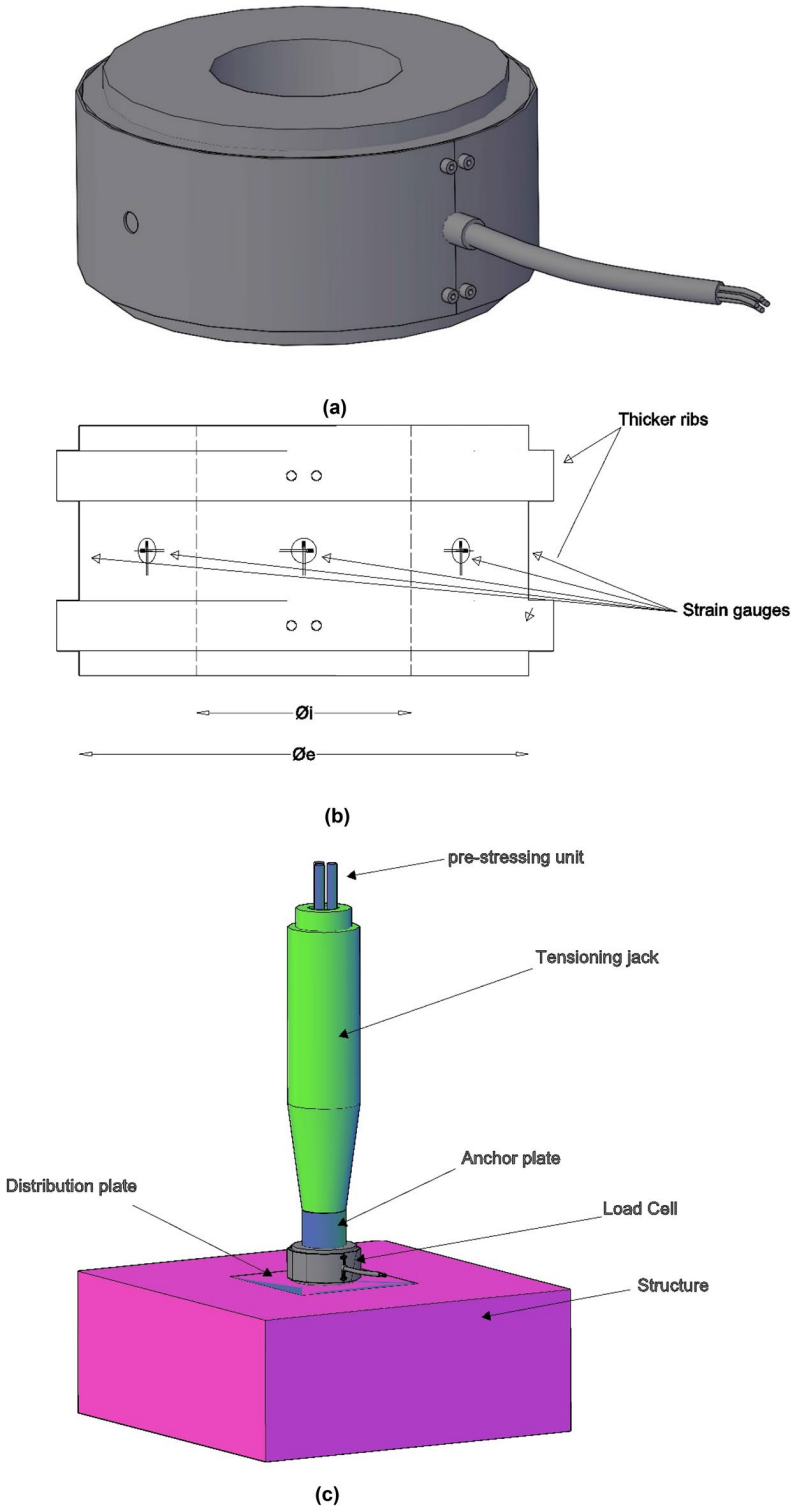
Design of type load cell for stress monitoring in prestressing units: (**a**) example of type load cell; (**b**) profile of type load cell; (**c**) example of installation in a 3-strand prestressing unit.

To ensure an accurate measurement of the total stress transmitted by the bridge stay or prestressing unit, the load cell must be provided with a sufficient depth “h” to uniform the stress concentration induced by the unequal support of the load cell on the distribution plate in the structure. For the study of the optimal edge to be provided to the load cell, the authors carried out a parametric study using a finite element model run with the structural calculation program Midas Civil 2022 v1.2 (http://www.simulsoft-ingenieros.es/nuestras-soluciones/midas-civil/midas-civil-descargas/) (Fig. 10). In the different Finite Element Models (FEM) used by the authors in the parametric study, the device under analysis is loaded with a uniform pressure (the anchor plate of the stays or prestressing units is a machined element, with a perfectly flat contact face, as is the contact face of the load cells), while on the other hand it is supported punctually on the bottom face (the distribution plate in the structure normally has a non-uniform surface, with depressions and bulges), causing stress concentration in the support areas. The result of this parametric study established that, to ensure accurate measurement, the minimum edge of the load cell should be set at a height equivalent to half the outer diameter of the ring forming its cross-section (h ≥ ∅/2). The load cells designed for the monitoring of stresses in the stay cables of the Tajo Bridge were designed by the authors following the indications provided by the results of this parametric study. These devices were tested in a press at the Structures Laboratory of the University of Cantabria with a load capacity of 3000 kN. The tests were carried out with different configurations of non-uniform supports and eccentric loads, obtaining a correct reading in the device, invariable with the test conditions. The inside diameter of the load cell shall be set by the prestressing unit or the bridge stay in such a way as to allow passage through the load cell (Fig. 11). Once the inside diameter has been established, the load cell shall be sized with an outside diameter that ensures that the mean normal stress in the load cell centre ring is less than or equal to half the yield strength of the material from which the load cell is made, see Eq. (3).

3 $$ \emptyset_{e} = \sqrt {\left( {\frac{{\text{N}}}{{\frac{{f_{y} }}{2}}} + \frac{\pi }{4} \cdot \emptyset_{i}^{2} } \right) \cdot \frac{4}{\pi }} $$

where ∅_e_ = load cell outer diameter; N = design load of the bridge stay; f_y_ = yield strength of the load cell material; ∅_i_ = load cell inside diameter.

**Figure 12:**
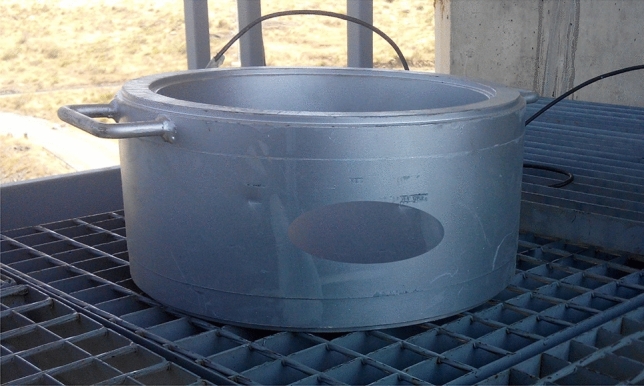
Example of a load cell to control the total stress experienced by the stay cables of a bridge.

In order to make the load cell sufficiently robust, it was decided to provide it with a thicker rib at the upper and lower ends of the load cell body. The central part of the load cell edge, on the outer perimeter of which the instrumentation is located, is covered by a metal jacket anchored to the upper and lower ribs. Subsequently, the gap between the metal jacket and the load cell body is injected with a resin that protects the instrumentation against impacts and atmospheric agents (Fig. 12). These devices are used in situ and, in the case of bridge stay cables, can weigh more than 100 kg and therefore require the use of lifting aids to handle them, and it is normal for them to be subjected to shocks during handling.

#### Discussion

**Figure 13:**
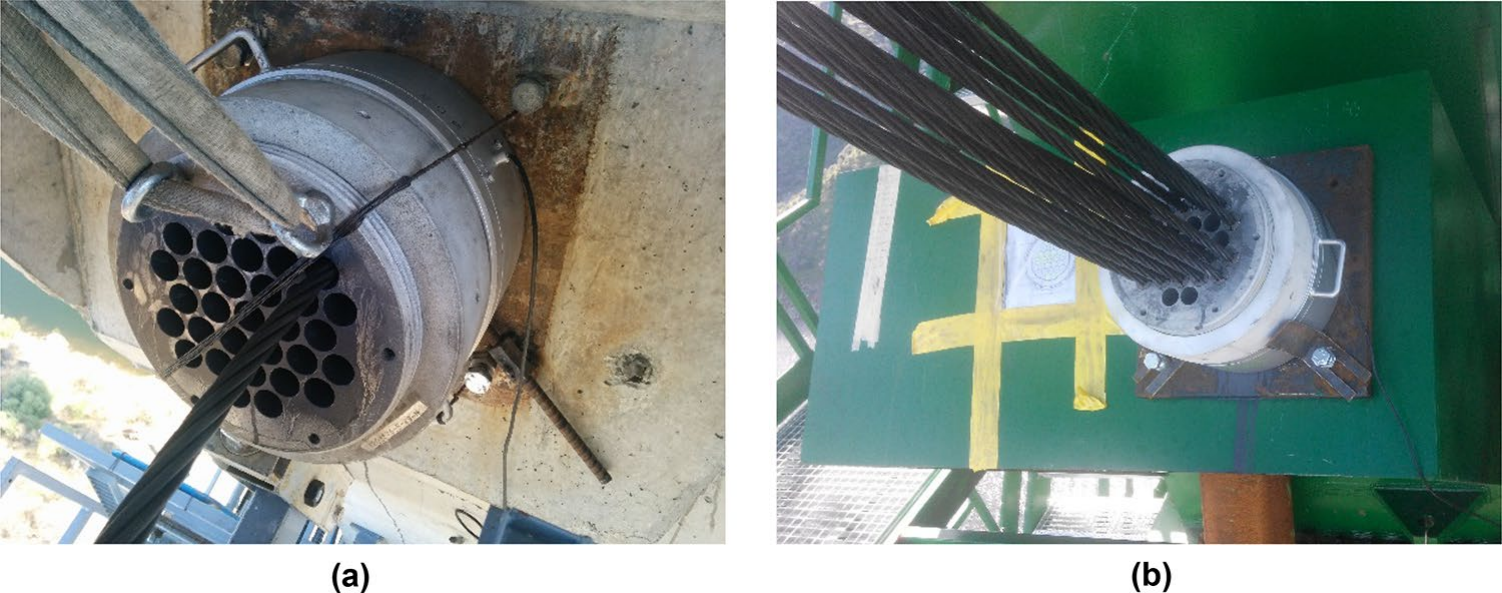
Tajo Bridge load cells: (**a**) load cell installation on cable-stayed pile; (**b**) load cell installation on cable-stayed tower.

**Figure 14:**
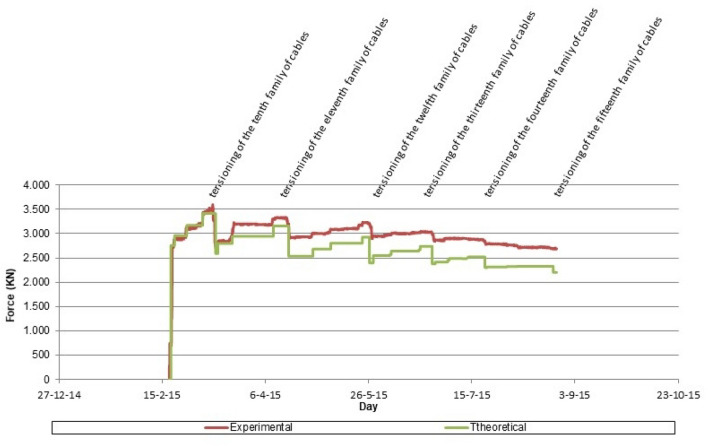
Force evolution in the ninth family of suspension stay cables.

Three techniques were simultaneously employed to monitor the bridge stays: (a) installation of load cells on the active anchors (Fig. 13); (b) installation of unidirectional strain gauges on one of the strands composing the stays; (c) installation of piezoelectric accelerometers on the stays. The load cells designed by the authors to control the structural stresses of the suspension stay cables of the central span of the Tajo bridge had an inside diameter ranging from 200 mm for the least loaded cables (2000 kN) to 500 mm for the most loaded cables (5500 kN) (Fig. 14). These devices made it possible to detect various structural phenomena inherent to the construction process. These include: (1) the monitoring of stress variations in the bridge stay, originated by daily thermal fluctuations, with force increases in the order of 150 kN for daily temperature variations of 30 K (30 °C); (2) the evaluation of stress variations derived from the concreting process of successive segments, with force oscillations that could range from 200/300 kN in the stays closest to the newly concreted segment, to values lower than the daily variations in the most distant stays; (3) the analysis of the force variation associated with the stressing of different families of cables, with force decreases of more than 500 kN in the families of stay cables closest to the loaded stay cable family and smaller than the daily force variations in the most distant families; (4) the detection of force variations resulting from load readjustment operations in the suspension stay cables. Figure 14 shows the contrast between the theoretical calculation values and the experimental values provided by the load cell installed on the instrumented stay cable of the ninth family of suspension stays of the north half-arch.

**Figure 15:**
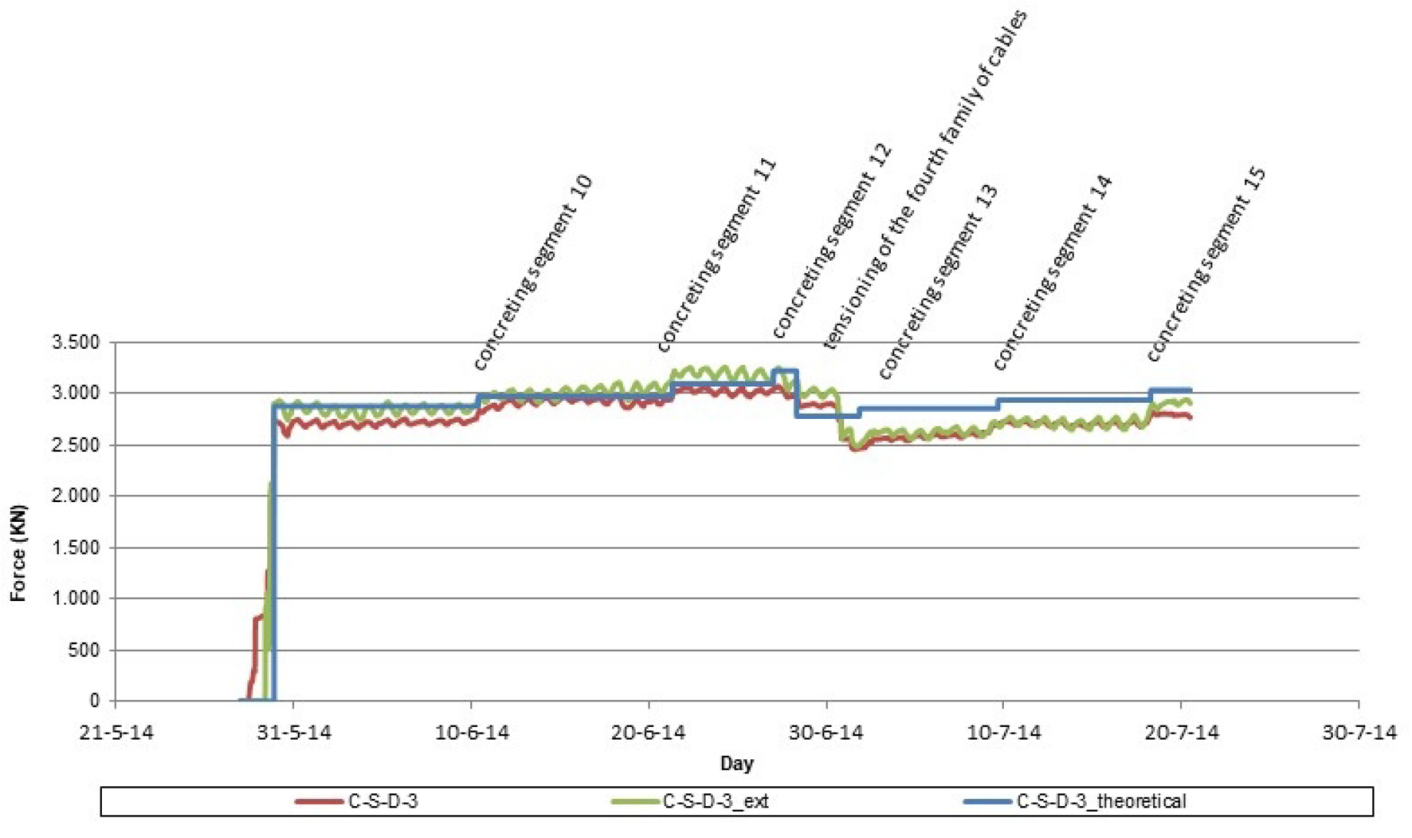
Stress evolution in the third family of suspension stay cables.

**Figure 16:**
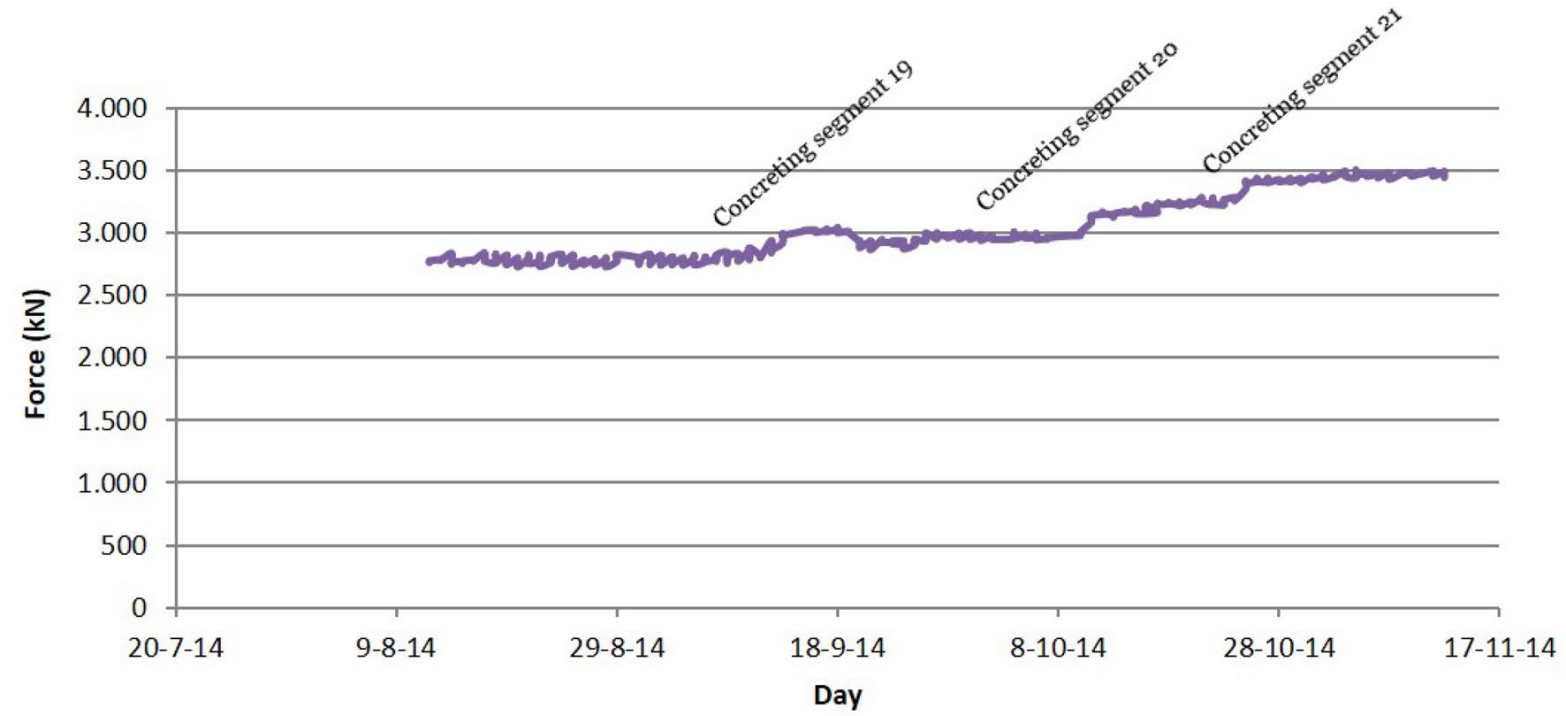
Stress evolution in the provisional stay instrumented by the installation of a piezoelectric accelerometer.

The simultaneous instrumentation of the first 4 families of stay cables by means of load cells and unidirectional strain gauges made possible a double contrast in relation to the stress value experienced by these structural elements. Figure 15 shows the evolution of the stress value in the instrumented stay cable of the third family of suspension stays of the southern half-arch: (C-S-D-3) value provided by the load cell; (C-S-D-3_ext) value provided by the extensometer; (C-S-D-3_theoretical) theoretical calculation value. In addition, it was decided to install a piezoelectric accelerometer on one of the temporary suspension stays. The purpose of this instrumentation is twofold: (1) to establish an early warning system for the possible appearance of undesirable eoroelastic phenomena; (2) to monitor the evolution of the force in the temporary suspension stay. Figure 16 shows the evolution of the force in the stay instrumented by means of the piezoelectric accelerometer.

### New synchronised multi-strain gauge load cell network for the monitoring of short prestressing units

**Figure 17:**
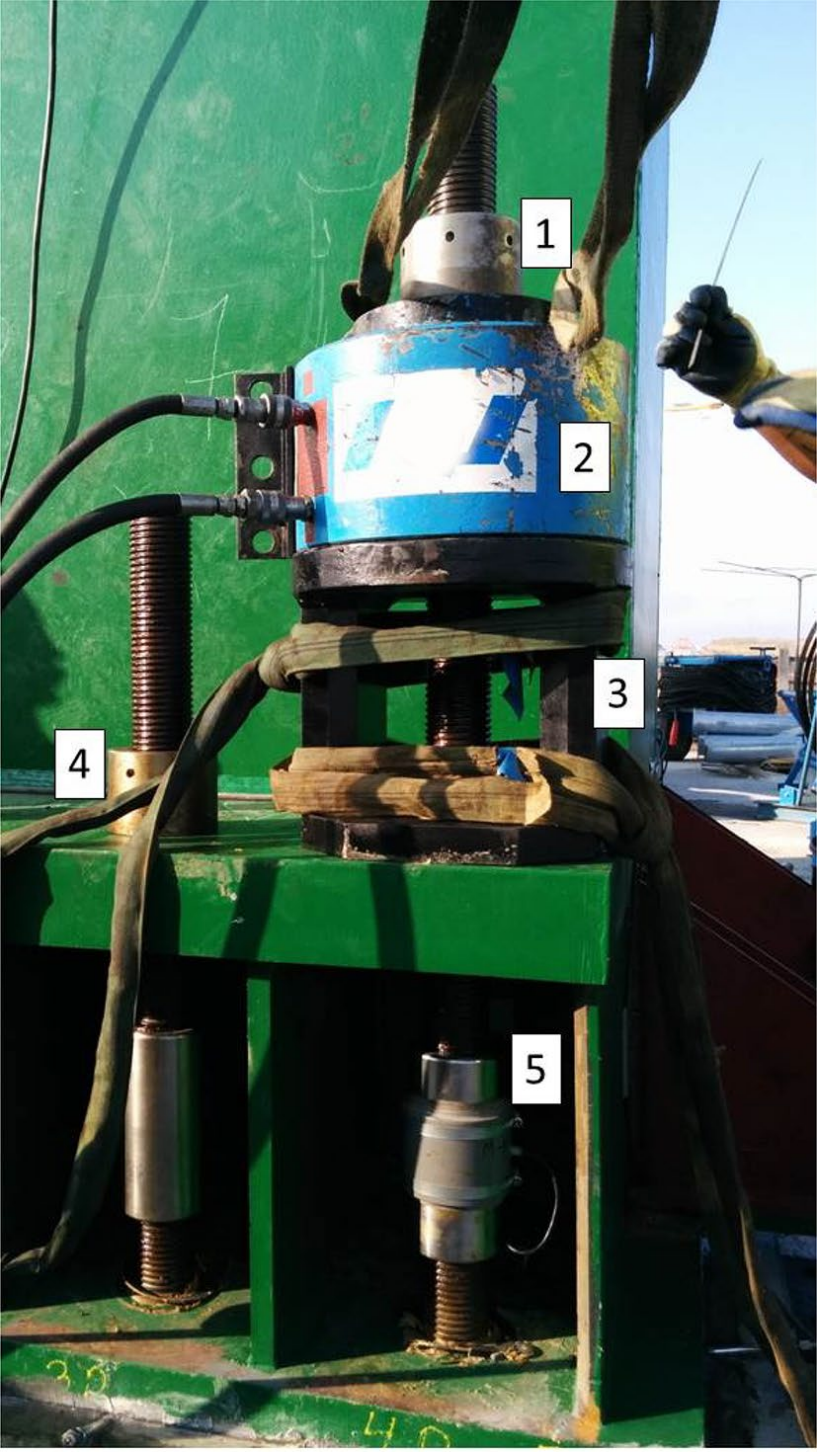
Macalloy bar prestressing operation: (1) reaction nut; (2) hollow tensioning jack; (3) tensioning bridge; (4) anchor nut; (5) strain gauge load cell.

Each temporary stay tower of the Tajo Bridge is anchored to the deck with a total of 16 prestressed Macalloy bars. Each of the Macalloy bars was tensioned with a load of 3,000 KN, giving a total prestressing stress in the tower-deck connection of 48,000 KN. The Macalloy bars are loaded with a hollow tensioning jack. To tighten the anchor nut of the Macalloy bar, a tensioning bridge must be provided between the hollow tensioning jack and the distribution plate at the base of the temporary stay tower. Finally, at the top of the hollow tensioning jack, a reaction nut is required to transmit the pre-stressing force exerted by the hollow tensioning jack to the Macalloy bar (Fig. 17). For the correct functioning of the prestressed connection between the stay towers and the deck, it is necessary to guarantee a uniform contact between the deck, made of pre-stressed concrete, and the stay tower, made of structural steel. For this purpose, an epoxy resin of fluid consistency was injected at the junction of the two structural elements. The authors proposed a synchronized multi-strain gage load cell network in each stay tower as a natural extension of the simple design for individual load cells (M1, M2, M3 and M4). The device designed by the authors for monitoring Macalloy bar stresses consisted of a Macalloy bar joint sleeve, instrumented by 4 bi-directional strain gages connected together by a complete electronic Wheatstone bridge assembly^26–28^. To obtain the relationship between the deformation experienced by the device and the force transmitted to the Macalloy bar, it is necessary to calibrate the device on a loading gantry. This process allows the force/strain curve of the device to be obtained and the linearity of this relationship to be checked for load values within the working range of the Macalloy bars to be instrumented (Fig. 14). Each bar was tensioned in 4 phases, each corresponding to a load of 25%, 50%, 75% and 100% strain. The pre-stress-free length of the Macalloy bars is given by the length between their passive anchorage in the cross section of the deck and the active anchorage at the support of the stay tower legs. This length is slightly more than 3 m, exactly 3.321 m, which means that if at the moment of tightening of the nut a settlement of 1 mm occurs, this settlement would cause an instantaneous prestressing loss of approximately 10% of the tensioning load, Eq. (4). The characterization of the prestressing losses in the different phases, regularization, nut tightening and creep, was one of the objectives that led to the approach of structural monitoring of the prestressed connection between the bridge deck and the temporary stay tower.

4 $$\Delta {\text{F}}=\frac{\Delta {{\text{L}}}_{{\text{t}}}\cdot \Omega \cdot {\text{E}}}{{{\text{L}}}_{{\text{t}}}}=\frac{1{\text{mm}}\cdot 4185{{\text{mm}}}^{2}\cdot 205\cdot {10}^{3}{\text{MPa}}}{\mathrm{3321mm}}=258\mathrm{ kN}=8.6\mathrm{\%Ft}$$

where: ΔF = prestressing loss; ΔLt = bar length increment (settlement); Ω = bar cross-section; E = modulus of elasticity of steel (Macalloy 1030); Lt = prestressing free length.

#### Discussion

**Figure 18:**
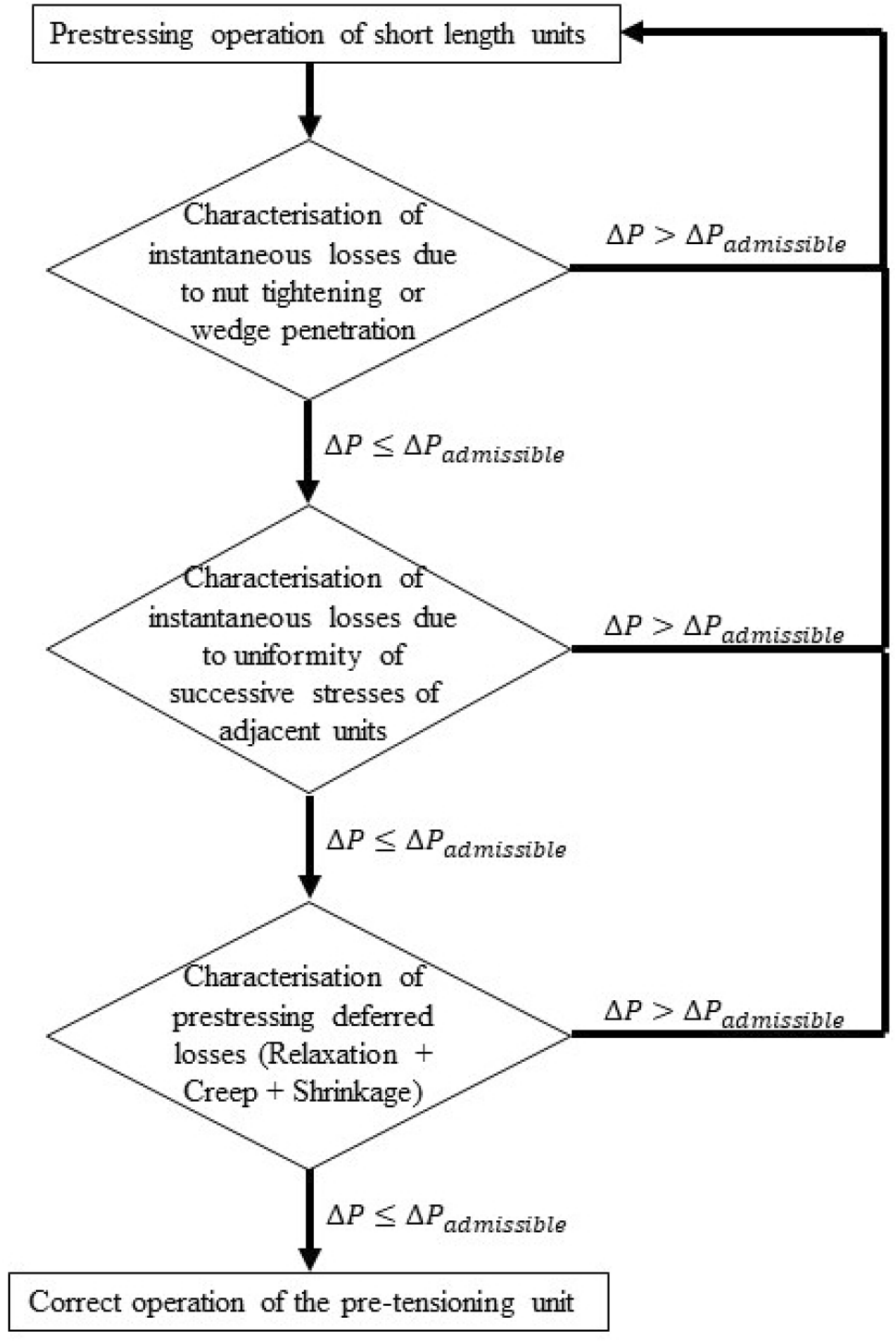
Flow chart for the correct execution of the tensioning operation of short length prestressing units.

**Figure 19:**
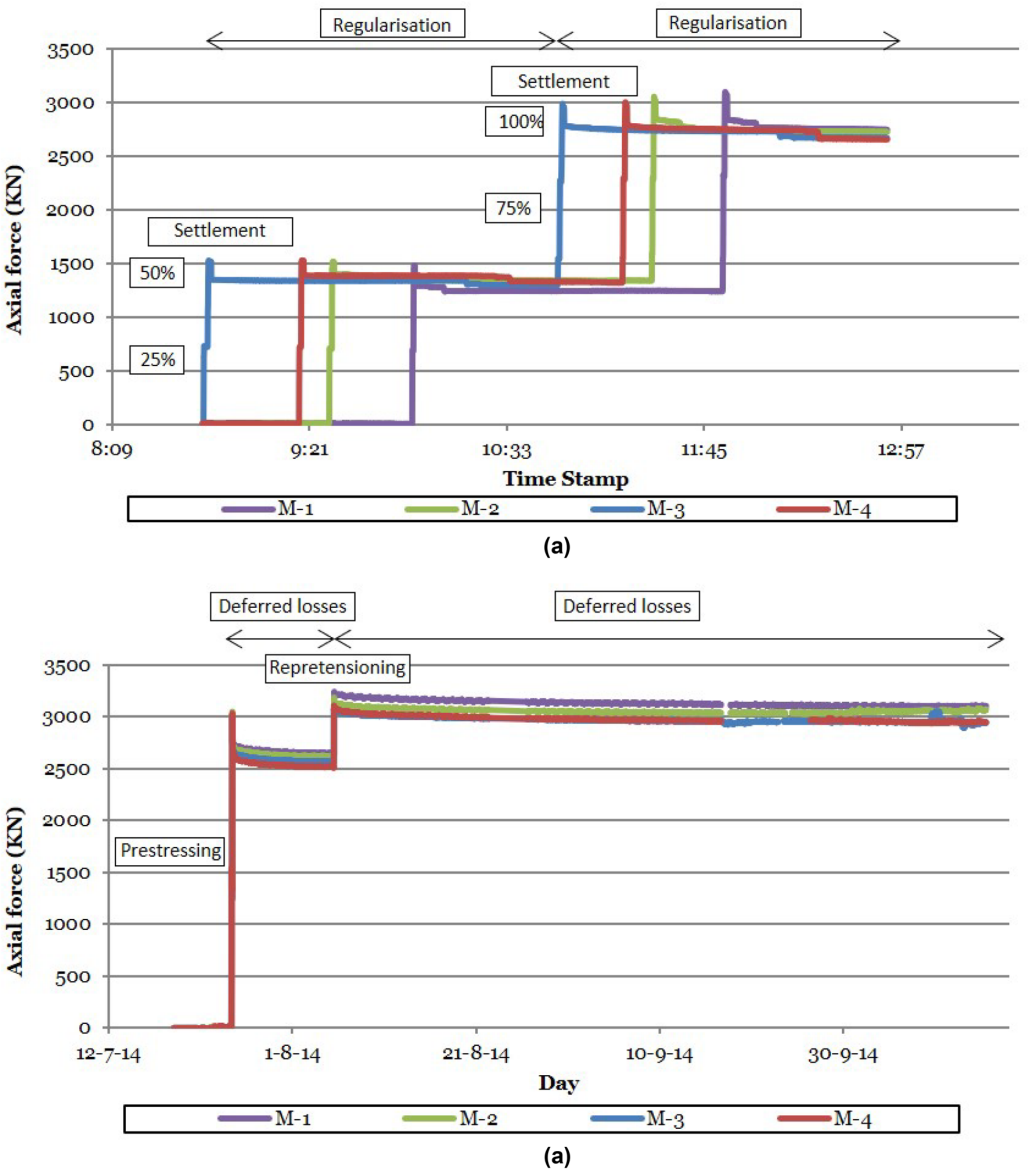
Prestressing stress evolution in Macalloy bars: (**a**) instantaneous prestressing losses; (**b**) deferred prestressing losses.

The system used ensures the correct prestressing of short prestressing units composed of multiple prestressed elements, allowing accurate quantification of the pre-stressing losses experienced by the prestressed connection. Figure 18 proposes the correct methodology to ensure the optimum prestressing force of a prestressed joint. This methodology is based on the instrumentation of the prestressed joint, and proposes 3 stages or control phases: (1) in a first stage, the instantaneous losses intrinsic to the prestressing unit typology (wedge penetration in the case of prestressing strands and nut tightening in the case of prestressing bars) are analysed; (2) in a second stage, the instantaneous losses due to force regularisation by the successive stressing of the prestressing units composing the joint are analysed; (3) and in a third stage, the deferred losses experienced by the prestressed joint are analysed. The monitoring of the prestressed joint makes it possible to quantify these prestressing losses, so that if in any of these control phases losses greater than the admissible prestressing loss are detected, the cycle is returned to the beginning and the prestressed joint is re-stressed. The instantaneous pre-stressing losses during the prestressing operation of the Macalloy bars reached an average value of 176 KN, representing 6% of the total prestressing stress, while the deferred losses experienced by the prestressed joint during the 11 days following the prestressing operation represented a prestressing force loss of 120 KN, 4% of the total prestressing force. By instrumenting the prestressed joint through the installation of strain gauge load cells on the Macalloy bars, it was possible to plan the re-stressing operation of these bars so that, once the instantaneous and deferred prestress losses were experienced, the pre-stressed joint of the stay tower was tensioned to its theoretical design value of 3000 kN/bar. Figure 19 shows the evolution of the prestressing stress in the prestressed connection between one of the temporary stay towers of the Tajo Bridge and the deck. Graph (a) shows the evolution of the tension in the 4 prestressing bars instrumented during the stressing of the 16 prestressing bars that make up the connection. In this case, the stressing of each bar was carried out in 4 phases corresponding to 25, 50, 75 and 100% of the design prestressing force. As can be seen in the graph, when 50% of the prestressing force is reached in a bar, the rest of the prestressing bars that make up the connection are stressed, producing an initial regularisation of stresses due to the successive stressing of the prestressing bars. Once all the bars that make up the joint are at a force corresponding to 50% of the prestressing force, the load on each prestressing bar is increased to 100% of its design force. This graph shows the instantaneous losses due to the tightening of the nuts and the instantaneous losses due to the regularisation of the stresses. Graph (b) shows the evolution of the prestressing force after completion of the tensioning operation, the retensioning of the prestressed joint 15 days after the first tensioning, because the prestressing losses exceeded the admissible losses, and the evolution of the prestressing force after completion of the retensioning operation of the prestressed joint.

## Conclusions

Through this paper, the authors aim to give an overview of the different monitoring systems currently used for stress monitoring control in bridge stays and prestressing units during the bridge construction phase, emphasizing the benefits of their integration with the domain knowledge provided by experts, enabling innovative techniques to control safety during specific construction procedures (and dismantling also) based on technologies who enjoy the confidence of construction sector.

As previously explained, load cells for active anchors have the advantage of being a robust and precise solution and their installation on stay cables during the successive cantilever construction processes allows to monitor the correct execution of the process and to plan the successive load adjustment operations on the stay cables if necessary. On the other hand, they have the disadvantage of being unwieldy and heavy and require installation prior to the stay cable one. The authors are proposing here a new load cell design consisting of a metal ring instrumented with strain gages and present the results of a parametric study to determine the minimum edge of the device (h ≥ ∅/2) to achieve more adequate accuracy in stress measurement. A formulation is also proposed to determine the cross-section of the device, which allows it to function correctly. The results of the use of the proposed device in the Tajo bridge construction process are presented which involves the validation of the new design under real and critical conditions.

In addition to this, a new synchronized multi-strain gage load cell network allows monitoring the tensioning operation in short prestressing units, quantifying the prestressing losses experienced by these structural elements and ensuring the work of the prestressed joint at the designated prestressing value. A flow chart is presented detailing the different control points for the correct execution of the loading of short prestressing units. The results of the use of this technique in the prestressing units that materialised the connection between the steel structure of the temporary stay towers and the prestressed concrete deck of the Tajo Bridge are presented. Thanks to the use of the technique proposed by the authors, it was possible to quantify the prestressing losses, which exceeded 10%, and to plan a new stressing operation that allowed the prestressed connection to be left with the design tension for its correct operation.

## Data Availability

The data that support the findings of this study are available from the corresponding author, Gaute A., upon reasonable request.
